# Amyloid beta and diabetic pathology cooperatively stimulate cytokine expression in an Alzheimer’s mouse model

**DOI:** 10.1186/s12974-020-1707-x

**Published:** 2020-01-28

**Authors:** Sitara B. Sankar, Carmen Infante-Garcia, Laura D. Weinstock, Juan Jose Ramos-Rodriguez, Carmen Hierro-Bujalance, Cecilia Fernandez-Ponce, Levi B. Wood, Monica Garcia-Alloza

**Affiliations:** 1 Wallace H. Coulter Department of Biomedical Engineering, Georgia Institute of Technology, Atlanta, GA 30332 USA; 2 Division of Physiology, School of Medicine, Universidad de Cadiz, Instituto de Investigacion Biomedica de Cadiz (INIBICA), Cadiz, Spain; 3 Instituto de Investigación e Innovación Biomédica de Cádiz (INiBICA), Cádiz, Spain; 4 Departamento de Fisiología, Facultad de Ciencias de la Salud, Universidad de Granada, Granada, Spain; 5 Área de Inmunología, Facultad de Medicina, Universidad de Cádiz, Cádiz, Spain; 6 George W. Woodruff School of Mechanical Engineering and Parker H. Petit Institute for Bioengineering & Bioscience, Georgia Institute of Technology, 315 Ferst Dr, Rm 3303, Atlanta, GA 30332 USA

**Keywords:** Pre-diabetes, Type 1 diabetes (T1D), Type 2 diabetes (T2D), Cytokine profile

## Abstract

**Background:**

Diabetes is a risk factor for developing Alzheimer’s disease (AD); however, the mechanism by which diabetes can promote AD pathology remains unknown. Diabetes results in diverse molecular changes in the brain, including dysregulation of glucose metabolism and loss of cerebrovascular homeostasis. Although these changes have been associated with increased Aβ pathology and increased expression of glial activation markers in APPswe/PS1dE9 (APP/PS1) mice, there has been limited characterization, to date, of the neuroinflammatory changes associated with diabetic conditions.

**Methods:**

To more fully elucidate neuroinflammatory changes associated with diabetes that may drive AD pathology, we combined the APP/PS1 mouse model with either high-fat diet (HFD, a model of pre-diabetes), the genetic db/db model of type 2 diabetes, or the streptozotocin (STZ) model of type 1 diabetes. We then used a multiplexed immunoassay to quantify cortical changes in cytokine proteins.

**Results:**

Our analysis revealed that pathology associated with either db/db, HFD, or STZ models yielded upregulation of a broad *profile* of cytokines, including chemokines (e.g., MIP-1α, MIP-1β, and MCP-1) and pro-inflammatory cytokines, including IL-1α, IFN-γ, and IL-3. Moreover, multivariate partial least squares regression analysis showed that combined diabetic-APP/PS1 models yielded cooperatively enhanced expression of the cytokine profile associated with each diabetic model alone. Finally, in APP/PS1xdb/db mice, we found that circulating levels of Aβ1-40, Aβ1-42, glucose, and insulin all correlated with cytokine expression in the brain, suggesting a strong relationship between peripheral changes and brain pathology.

**Conclusions:**

Altogether, our multiplexed analysis of cytokines shows that Alzheimer’s and diabetic pathologies cooperate to enhance profiles of cytokines reported to be involved in both diseases. Moreover, since many of the identified cytokines promote neuronal injury, Aβ and tau pathology, and breakdown of the blood-brain barrier, our data suggest that neuroinflammation may mediate the effects of diabetes on AD pathogenesis. Therefore, strategies targeting neuroinflammatory signaling, as well as metabolic control, may provide a promising strategy for intervening in the development of diabetes-associated AD.

## Introduction

Alzheimer’s disease (AD) is the most common cause of dementia [1]. It is characterized neuropathologically by the progressive appearance of senile plaques composed of aggregated amyloid beta (Aβ), followed by microglial and astrocytic immune responses, formation of neurofibrillary tangles, neuronal dystrophy, and neuronal death [2, 3]. While aging remains the main risk factor for AD, the association between type 2 diabetes (T2D) and AD is particularly robust, as evidenced by epidemiological studies and supported by molecular, functional, and clinical data [4–7]. Also, prediabetes, as an initial step to later develop T2D, has been associated with AD [8, 9], and previous studies have suggested a role for type 1 diabetes (T1D) in AD [10, 11]. At the molecular level, some relevant links between diabetes and AD have been found. Among others, (i) insulin receptors are highly expressed in CNS regions relevant for cognition and memory, such as the cortex and hippocampus, and insulin has been shown to influence memory [12, 13]; (ii) Aβ oligomers induce insulin resistance in hippocampal neurons, suggesting a type of brain diabetes that may link Aβ to memory deficits [14]; and (iii) neurovascular damage impairs Aβ clearance along interstitial-fluid drainage pathways [15, 16], and both high Aβ and high glucose can compromise vascular health. In fact, the coexistence of metabolic diseases in mouse models of AD exacerbates AD hallmarks and memory deficits in these mice, as well as the inflammatory process associated with AD, prediabetes, and diabetes [11, 17]. Further, anti-diabetic drugs protect cognitive functions in AD mouse models and AD patients [18, 19].

Although the relationship between diabetes and AD might be attributed to some or all of the aforementioned factors [20], the ultimate cause of AD remains elusive. Individuals with unusually high levels of Aβ and/or neurofibrillary tangles do not necessarily suffer from cognitive decline or neuronal loss, and these resilient cases differ from AD patients in that they exhibit a reduced level of glial activation markers GFAP and Iba-1 [21], thus suggesting a role for the brain’s immune system in AD. The inflammatory response to AD is initiated by microglia, which migrate towards Aβ plaques and surround them. Microglial cells then secrete pro-inflammatory cytokines, including IL-1, IL-6, and TNF-α, as well as chemokines, such as MIP-1α and MCP-1, that attract astrocytes to envelop the plaques [22, 23]. Whether these glial responses are protective or deleterious is a matter of debate. One line of thought is that microglial and astrocytic responses reflect a protective immune function aimed at sequestering and degrading plaques. However, there is mounting evidence that glial responses to secreted cytokines and Aβ contribute to AD pathogenesis by producing factors that can be neurotoxic, like nitric oxide. Moreover, certain cytokines, such as TNF-α, IFN-γ, IL-6, and VEGF in combination with Aβ also contribute to neuronal death [24, 25], and IL-6 can upregulate amyloid precursor protein synthesis and processing, thereby accelerating plaque formation and disease progression [26]. However, due to the multiple functions and cross-talk of different cytokines, univariate analyses of cytokines do not provide a holistic picture of the neuroinflammatory microenvironment in pathological or control conditions. In light of these challenges, we have previously used multivariate analysis to correlate brain cytokine profiles with AD severity and to identify previously unnoticed cytokines that may play specific roles in disease progression [25]. We also used this type of analysis to identify distinct profiles of cytokines that may distinguish patients *resilient* to AD pathology from both controls or patients with AD [27].

In the present study, we used multivariate analysis tools to profile brain cytokine protein expression in the APPswe/PS1dE9 (APP/PS1) mouse model of AD amyloid pathology. We studied the APP/PS1 model alone or in combination with either a prediabetic state induced by a high-fat diet (APP/PS1-HFD), a well-established T1D induced by streptozotocin (APP/PS1-STZ), or a well-established T2D induced by crossing APP/PS1 mice with the classic T2D mouse model db/db (APP/PS1xdb/db). We describe correlations found between cytokine expression and pathological hallmarks and identify cytokines that may dissect specific aspects of these disease combinations, opening the door to establish different cytokine profile signatures associated with AD, prediabetes, T2D, or the combination of these commonly associated diseases.

## Material and methods

### Animals and treatments

APP/PS1 mice were obtained from the Jackson Laboratory (Bar Harbor, ME, USA) [28, 29]. Prediabetes was induced by HFD (60% Kcal from fat, OpenSource, New Brunswick, NJ, USA) ad libitum administration to APP/PS1 mice from 4 to 26 weeks of age, as previously described [30]. All other groups were fed with regular diet (SAFE A04. Augy, France). T1D diabetes was induced in wildtype and APP/PS1 mice at 18 weeks of age by intraperitoneal (i.p.) injection of streptozotocin (STZ, 40 mg/kg) for five consecutive days. STZ-treated mice were aged to 26 weeks. db/db mice were used as a model of T2D, and mixed AD-T2D mice were obtained by crossing db/db with APP/PS1 mice as previously described [11] and were also aged to 26 weeks of age. Both males and females were included in the study, as noted in the figure legends [11, 17, 30, 31]. Animals were sacrificed by intraperitoneal pentobarbital overdose (120 mg/kg). Left hemispheres were dissected and flash frozen and stored at − 80 °C until used. Right hemispheres were fixed in PFA 4%, and 30 μm coronal sections were obtained on a cryostat (Microm HM525, Thermo Scientific, Spain).

All experimental procedures were approved by the Animal Care and Use Committee of the University of Cadiz and Junta de Andalucía (09-07-15-282) in accordance with the Guidelines for Care and Use of experimental animals (European Commission Directive 2010/63/UE and Spanish Royal Decree 53/2013).

### Metabolic assessment

Body weight and postprandial glucose levels and insulin levels were determined in all mice under study at 26 weeks of age as previously described [17, 18]. Metabolic assessment was performed in the morning (8:00–11:00 a.m), immediately before sacrifice. Blood was collected at sacrifice. Glucose levels were measured with a glucometer Optium Xceed (Abbott, USA). Plasma was separated by centrifugation (7 min at 6500 rpm), and insulin levels were measured by ultrasensitive insulin ELISA according to the manufacturer’s indications (Mercodia Inc., Winston Salem NC) [17, 18].

### Aβ levels

Soluble and insoluble Aβ40 and Aβ42 levels were quantified by colorimetric ELISA kits (Wako, Japan) as previously described with minor modifications [17]. Somatosensory cortex (5–10 mg) was homogenized in 50 μl of lysis buffer (Pierce™ IP Lysis Buffer, cod. Cat 87787 Thermo Scientific, Spain) with Halt protease inhibitor cocktail 100x (cod. cat1862209 Thermo Scientific, Spain) and centrifuged at 14,500 rpm and 4 °C for 12 min. For soluble Aβ40 and 42 levels, supernatants were diluted 1:300 in H_2_Odd prior to running the ELISA. For insoluble Aβ levels, pellets were extracted with 65 μl of 70% formic acid and centrifuged at 14,500 rpm and 4 °C for 10 min. After neutralizing with 1 M Tris (pH 11), samples were diluted (1:10) in standard diluent from the ELISA kit. Blood samples were extracted immediately before sacrifice and centrifuged at 6500 RPM for 7 min. Plasma was collected and diluted 1:2 in ddH_2_O for quantification of Aβ40 and 42 via ELISA (Wako) according to the manufacturer’s protocols. Absorbance was measured spectrophotometrically at 450 nm (MQX200R2, Biotek instruments, Burlington VT, USA), and data were expressed as pmol/g tissue or pmol/L of plasma.

### Aβ and microglia immunostaining

Right hemisphere sections were selected at 1.5, 0.5, − 0.5, − 1.5, − 2.5, and − 3.5 mm from Bregma [32]. Sections were pretreated with formic acid (70%) and incubated with anti-Iba1 (Wako, Osaka, Japan) (1:1000) and anti-Aβ (4G8, Covance, Greenfield, IN, USA) (1:2000) antibodies at 4 °C in 0.5% BSA overnight. Alexa Fluor 594 and Alexa Fluor 488 (Molecular Probes, OR, USA) (1:1000) were used as secondary antibodies. A Laser Olympus U-RFL-T fluorescent microscope (Olympus, Japan) and MMIcellTools v.4.3 (Molecular Machines and Industries, Eching, Germany) software was used was used to for image acquisition. Senile plaque (SP) burden and microglia burden in proximity of (within 50 μm) and far from (> 50 μm) SP were measured using Image J software as previously described [17].

### Statistical analysis

One-way ANOVA followed by Tukey *b* test, Tamhane test, or Dunnett’s test as required were used. SPSS v.24 and GraphPad Prism 7 (GraphPad Prism, San Diego, CA) software was used for all statistical analysis. Outliers were removed using GraphPad Prism’s robust regression and outlier removal (ROUT) method.

### Luminex analysis of cytokines

Brain cortices were homogenized and lysed using a Bio-Plex cell lysis kit (BioRad, 171-304011), with the addition of protease inhibitor cocktail (Thermo Scientific Pierce, Spain) following the manufacturer’s directions. Lysates were centrifuged at 14,500 rpm and 4 °C for 12 min. Supernatants were stored at − 80 °C until used. Protein content was determined by Bradford assay [33]. Samples were normalized to 7.5 μg/μl in 0.5% bovine serum solution, and 50 μL of each sample was added to the Bio-Plex kit. Cytokine protein was quantified using the Bio-Plex Pro™ Luminex Cytokine panel (BioRad 10,014,905) and read out using a Bio-Plex Manager Software v 6.0 and Bio-Plex 200 system (Bio-Rad, Spain). Data were expressed in pg/mg total protein, by Bradford analysis [33]. G-CSF was excluded from analysis because it was not detectable above background.

### Partial least squares modeling

Partial least squares regression (PLSR) and PLS discriminant analysis (PLSDA) were conducted in MATLAB using the partial least squares algorithm by Cleiton Nunes (Mathworks File Exchange). All data were *z*-scored, and then directly inputted to the algorithm. For each PLSDA and PLSR analysis, an orthogonal rotation in the LV1-LV2 plane was used to choose a new LV1 that better separated groups or phenotype/*Y*-variable, respectively. A Monte Carlo subsampling of 80% of the samples with 1000 iterations without replacement was used to compute SDs for LV signals. To correct for sign reversals, each subsampled LV1 and LV2 was multiplied by the sign of the scalar product of the new LV and the corresponding LV from the total model. When ANOVA was used to analyze scores on both LV1 and LV2, orthogonality was checked for and the scalar product between the two components was ensured to be < 1 × 10^−15^.

## Results

### Metabolic alterations

Body weight, glucose, and insulin levels were used to quantify metabolism of each animal model at 26 weeks of age. Weight and metabolic measurements (insulin and glucose) were not significantly affected in APP/PS1 animals compared to wild-type mice (Additional file 1: Figure S1A). However, there is the possibility that more subtle alterations may be present [34], and we did not detect significant differences due to limited statistical power. In prediabetic mice (HFD and APP/PS1-HFD), body weight and insulin levels were increased, consistent with a prediabetic phenotype (Additional file 1: Figure S1A). T1D was induced by STZ treatment for five consecutive days starting at 18 weeks of age–8 weeks prior to assessment of metabolism and pathology. STZ and APP/PS1-STZ mice presented a modest reduction in body weight. Insulin levels were reduced in STZ-treated mice, and hyperglycemia was detected (Additional file 1: Figure S1A). T2D mice (db/db and APP/PS1xdb/db) were overweight and both plasma insulin and glucose levels were significantly increased (Additional file 1: Figure S1A).

### Amyloid pathology was altered in diabetic models

As previously observed, metabolic disease affected the kinetics of amyloid deposition in APP/PS1 mice. In particular, we observed that senile plaque (SP) burden was significantly reduced in STZ-treated APP/PS1 (T1D-AD) and in APP/PS1xdb/db (T2D-AD) mice compared to APP/PS1 mice [*F*_(3,21)_ = 11.81, **p < 0.01 vs. APP/PS1-STZ and APP/PS1xdb/db] (Additional file 1: Figure S1B). Similar changes were observed when we measured insoluble Aβ levels (Aβ40 [*F*_(3,18)_ = 5.66, **p < 0.01 vs. rest of the groups]; Aβ42 [*F*_(3,18)_ = 6.43, ^††^*p* < 0.01 vs. APP/PS1-HFD) (Additional file 1: Figure S1B). However, soluble Aβ levels were increased in APP/PS1xdb/db mice (Aβ40 [*F*_(3,18)_ = 16.12, **p < 0.01 vs. rest of the groups]; Aβ42 [*F*_(4,18)_ = 16.96, **p < 0.01 vs. rest of the groups]) (Additional file 1: Figure S1B).

### Microglial burden

Microglia burden was measured in close proximity to SP (< 50 μm) or far from SP (> 50 μm). An overall increase in microglia burden was observed in SP-free areas within T2D mice [*F*_(7,42)=_4.68, **p = 0.001 vs. control and APP/PS1] (Additional file 1: Figure S1C and D) whereas no differences were detected in proximity to SP [*F*_(7,21)_ = 1.98, *p* = 0.147] **(**Additional file 1: Figure S1C and D). Our observations are in line with previous studies from our lab analyzing microglia burden in these animal models [11, 17, 30, 31], and microglia appear to be more amoeboidal in the vicinity of senile plaques (Additional file 1: Figure S1D).

### STZ type 1 diabetic model stimulates cytokine production in APP/PS1 mice

**Fig. 1:**
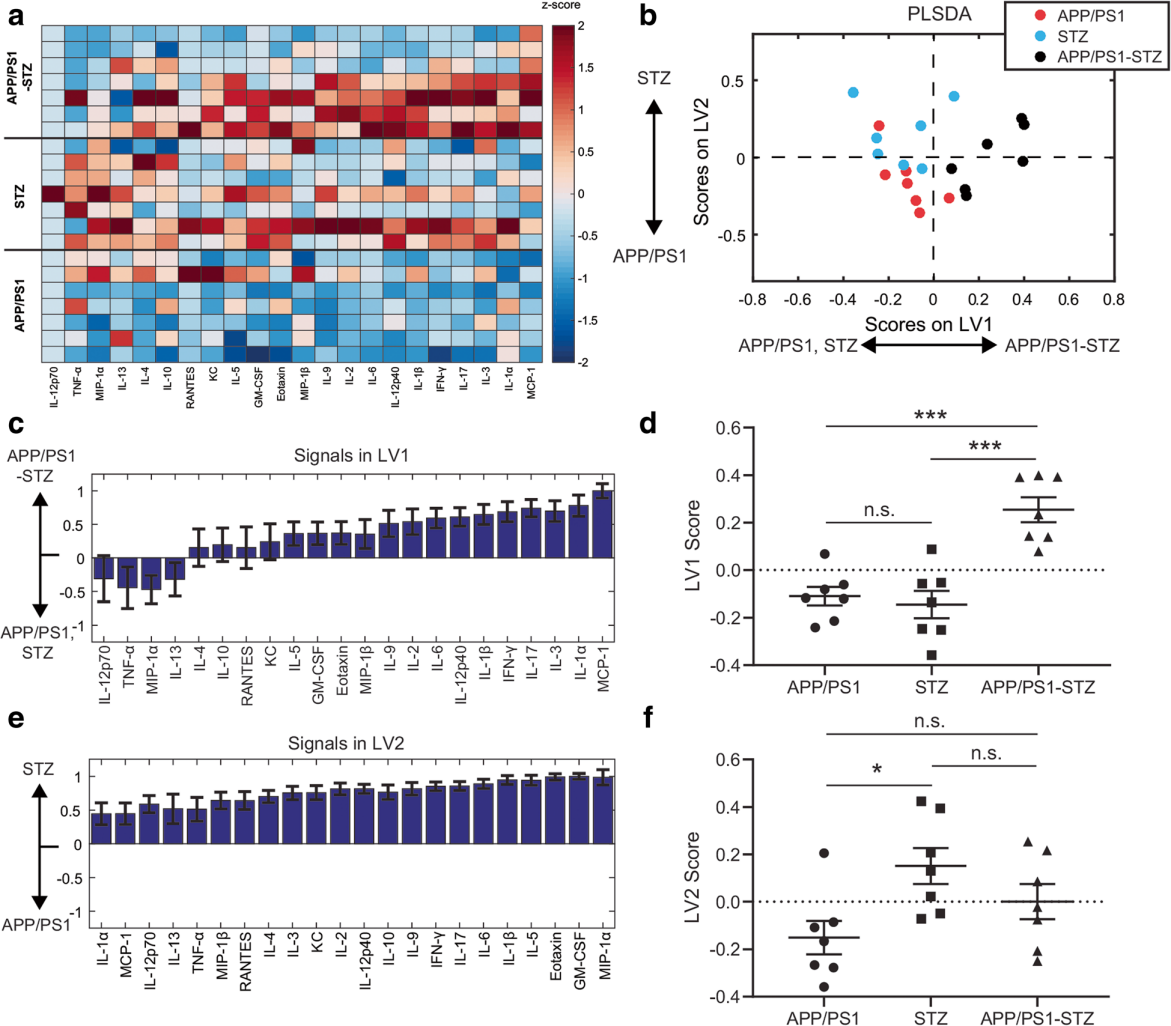
APP/PS1 pathology and STZ cooperatively promote cytokine expression. **a** Luminex analysis of 22 cytokines (columns, *z*-scored) expressed in the cortex of APP/PS1, STZ, and APP/PS1-STZ mice (each row is a cortex sample). **b** Partial least squares discriminant analysis (PLSDA) identified two profiles of cytokines, LV1 and LV2, that distinguished groups. LV1 separated APP/PS1-STZ mice (positive) from both APP/PS1 and STZ mice (negative). LV2 separated STZ mice (positive) from APP/PS1 mice (negative). **c** The weighted profile of cytokines representing LV1. Errors bars on each cytokine were computed by PLSDA model regeneration using iterative subsampling of 80% of samples (mean ± SD). **d** Scoring the data for each sample in **a** on LV1 revealed that combined APP/PS1-STZ pathology cooperatively increased the LV1 cytokine profile compared to either APP/PS1 or STZ pathology alone (***p < 0.001, Welch’s ANOVA with Dunnett’s T3 test). **e** The weighted profile of cytokines representing LV2. Errors bars on each cytokine were computed by PLSDA model regeneration using iterative subsampling of 80% of samples (mean ± SD). **f** Scoring the data for each sample in **b** on LV2 revealed that STZ is significantly upregulated on the LV2 cytokine profile compared to APP/PS1 (*p < 0.05, Welch’s ANOVA with Dunnett’s T3 test). Data were collected from 21 mice (16 M/12/F, STZ5M/2F, APP/PS1 3 M/4F, APP/PS1-STZ 4 M/3F)

Since STZ-induced T1D pathology reduced SP burden in APP/PS1 mice (Additional file 1: Figure S1B), we hypothesized that this alteration in pathology would be accompanied by an enhanced neuroinflammatory response in APP/PS1-STZ mice compared to APP/PS1 mice at the 26-week time point. To test this, we used Luminex analysis (Bio-Rad) to quantify protein expression of 22 cytokines/chemokines in mouse cortical tissues. G-CSF levels were under detection limits for the majority of the animals under study and G-CSF was thus excluded. Our analysis showed that STZ treatment induced robust cytokine expression in APP/PS1 mice compared to untreated controls (Additional file 1: Figure S2). Since we were primarily interested in the differences in cytokine expression of APP/PS1-STZ combined pathology compared to either APP/PS1 or STZ pathology alone, we represented the panel of cytokines in terms of their *z*-scores (mean subtracted and normalized to standard deviation) with respect to these three groups (Fig. 1a). To account for the multi-dimensional nature of our data, we used a partial least squares discriminant analysis (PLSDA) to identify composite profiles of cytokines, called latent variables (LV1 and LV2), that distinguished between groups, as we have done previously [25, 27, 35] (Fig. 1b). LV1 consisted of a weighted profile of cytokines that together distinguished APP/PS1-STZ combined pathology mice from either pathology alone (Fig. 1c, d), while LV2 distinguished STZ from APP/PS1 pathology (Fig. 1e, f**)**. While scoring the individual samples on LV2 revealed that STZ-induced T1D pathology significantly upregulated that cytokine profile compared to APP/PS1 pathology, more interestingly, scoring samples on LV1 revealed that APP/PS1 with STZ-induced T1D pathology robustly increased scores on the LV1 cytokine profile compared to either pathology alone (Fig. 1d). A similar trend was observed when analyzing each cytokine on an individual basis (Additional file 1: Figure S3). Importantly, the cytokine weights in LV1 identify those cytokines that most strongly discriminate between combined APP/PS1-STZ pathology and the other groups. The top cytokines on LV1, MCP-1, IL-1α, IL-3, and IL-17 all have strongly pro-inflammatory and chemotactic properties [36–41]. Therefore, these data indicate that the STZ-induced T1D condition contributes to an elevated pro-inflammatory environment in the context of amyloid pathology.

### Db/db T2 diabetic model cooperatively stimulates cytokine production in APP/PS1 mice

**Fig. 2:**
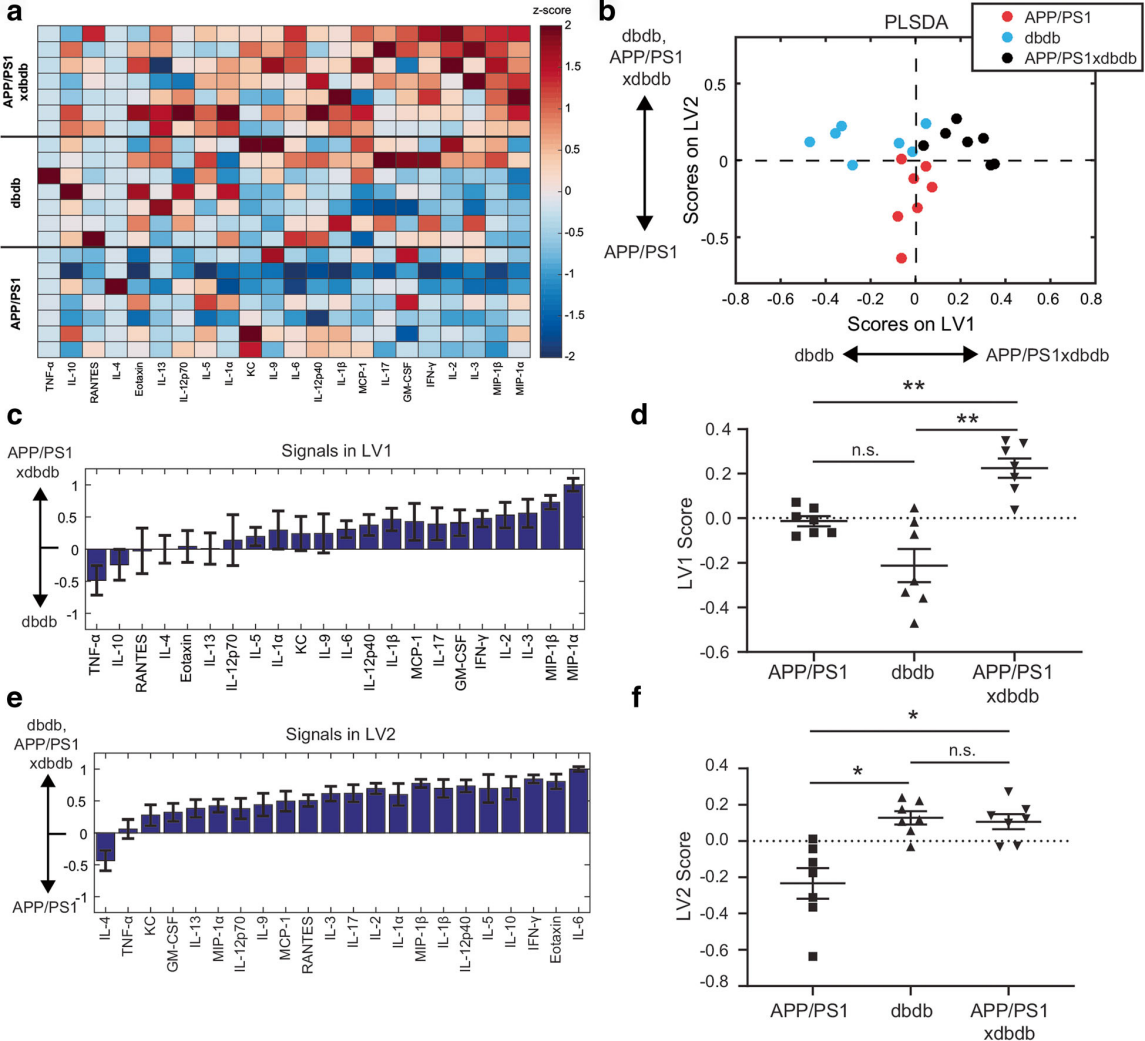
APP/PS1 and db/db pathologies cooperatively promote cytokine expression. **a** Luminex analysis of 22 cytokines (columns, *z*-scored) expressed in the cortex of APP/PS1, db/db, and APP/PS1xdb/db mice (each row is a cortex sample). **b** PLSDA identified two profiles of cytokines, LV1 and LV2, that distinguished groups. LV1 separated APP/PS1xdb/db mice (positive) from both APP/PS1 and db/db mice (negative). LV2 separated STZ mice (positive) from APP/PS1 mice (negative). **c** The weighted profile of cytokines representing LV1. Errors bars on each cytokine were computed by PLSDA model regeneration using iterative subsampling of 80% of samples (mean ± SD). **d** Scoring the data for each sample in **b** on LV1 revealed that combined APP/PS1xdb/db pathology cooperatively increased the LV1 cytokine profile compared to either APP/PS1 or db/db pathology alone (**p < 0.01, Welch’s ANOVA with Dunnett’s T3 test). **e** The weighted profile of cytokines representing LV2. Errors bars on each cytokine were computed by PLSDA model regeneration using iterative subsampling of 80% of samples (mean ± SD). **f** Scoring the data for each sample in **b** on LV2 revealed that APP/PS1xdb/db is significantly upregulated on the LV2 cytokine profile compared to db/db (*p < 0.05, Welch’s ANOVA with Dunnett’s T3 test). Data were collected from 21 animals (14 M/14F, APP/PS1 2 M/51F, db/db 3 M/5F, APP/PS1xdb/db 6 M/1F)

Since microglial burden and amyloid levels were also changed in APP/PS1xdb/db mice, we next asked whether or not cytokine expression would also be modulated in this T2D model. To test this, we again used Luminex analysis to quantify expression of cytokines in the cortex (Figs. 2 and Additional file 1: Figure S4). PLSDA analysis identified that APP/PS1, db/db, and combined APP/PS1xdb/db pathology were elevated on a profile of cytokines compared to controls (Additional file 1: Figure S4). Focusing our analysis to distinguish differences between the APP/PS1xdb/db combined pathology and each individual pathology, we identified two cytokine profiles, LV1 and LV2, that distinguished between groups (Fig. 2b). LV2 distinguished APP/PS1 from db/db diabetic pathology (Fig. 2b, e, f). Scoring samples on this profile revealed that animals with db/db pathology are elevated on this profile, regardless of the presence of APP/PS1 pathology (Fig. 2f). More interestingly, however, LV1 distinguished APP/PS1xdb/db mice from APP/PS1 pathology alone or db/db alone (Fig. 2b–d). Analysis of each cytokine on an individual basis showed a similar trend (Additional file 1: Figure S5). Like in the STZ T1D model, the most upregulated cytokines distinguishing APP/PS1xdb/db mice from either APP/PS1 or db/db mice had strongly chemotactic or pro-inflammatory properties (i.e., MIP-1α, MIP-1β).

### High-fat diet cooperatively stimulates cytokine production in APP/PS1 mice

**Fig. 3:**
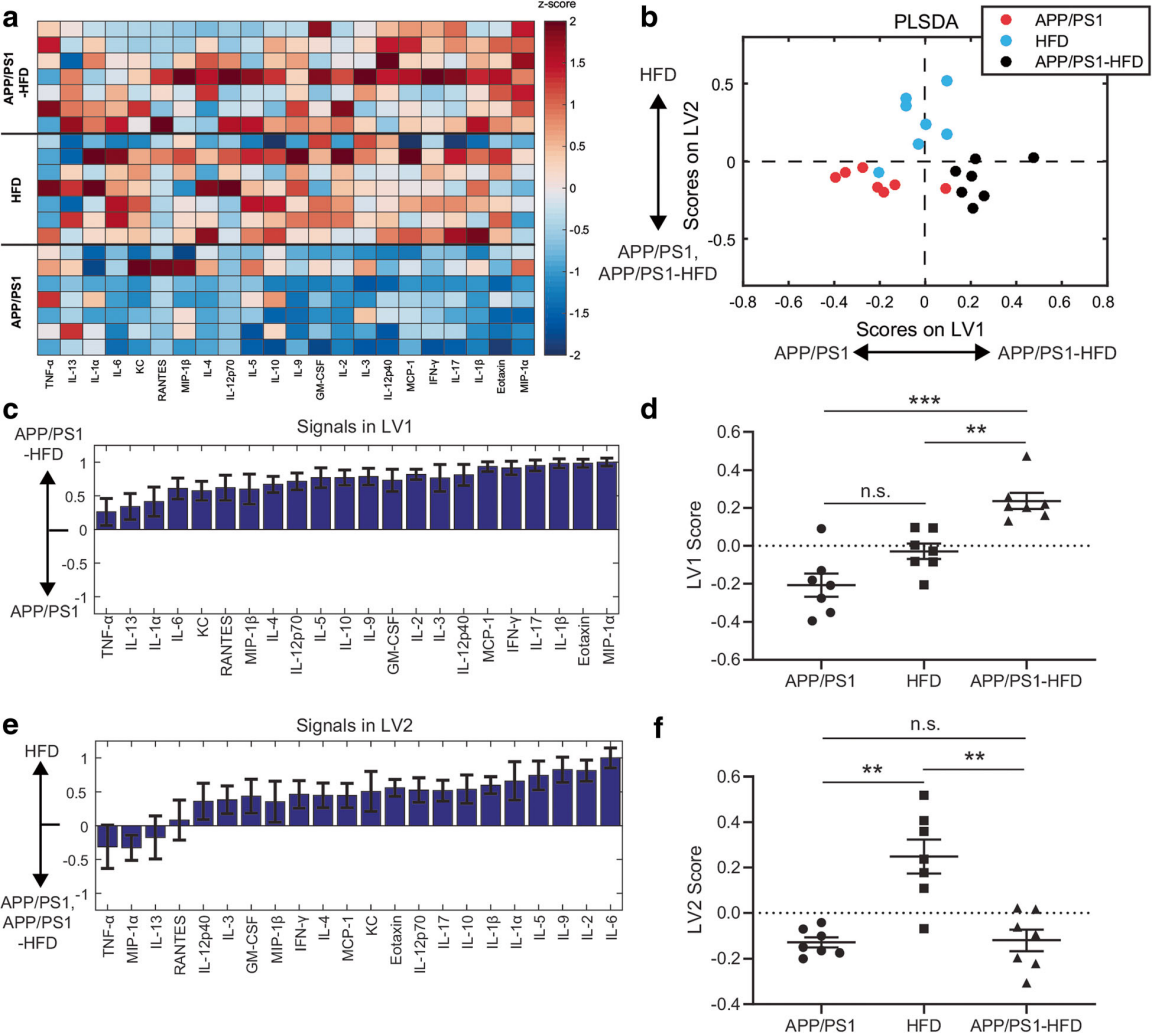
APP/PS1 pathology and high-fat diet cooperatively promote cytokine expression. **a** Luminex analysis of 22 cytokines (columns, *z*-scored) expressed in the cortex of APP/PS1, HFD, and APP/PS1-HFD mice (each row is a cortex sample). **b** PLSDA identified two profiles of cytokines, LV1 and LV2, that distinguished groups. LV1 separates APP/PS1-HFD mice (positive) from both APP/PS1 and HFD mice (negative). LV2 separates HFD mice (positive) from APP/PS1 and APP/PS1-HFD mice (negative). **c** The weighted profile of cytokines representing LV1. Errors bars on each cytokine were computed by PLSDA model regeneration using iterative subsampling of 80% of samples (mean ± SD). **d** Scoring the data for each sample in **a** on LV1 revealed that combined APP/PS1-HFD pathology cooperatively increased the LV1 cytokine profile compared to either APP/PS1 or db/db pathology alone (**p < 0.01, Welch’s ANOVA with Dunnett’s T3 test). **e** The weighted profile of cytokines representing LV2. Errors bars on each cytokine were computed by PLSDA model regeneration using iterative subsampling of 80% of samples (mean ± SD). **f** Scoring the data for each sample in **b** on LV2 revealed that HFD is significantly upregulated on the LV2 cytokine profile compared to both APP/PS1 and APP/PS1-HFD (**p < 0.01, Welch’s ANOVA with Dunnett’s T3 test). Data were collected from 21 animals (11 M/10F, HFD 4 M/3F, APP/PS1 3 M/4F, APP/PS1-HFD 4 M/3F)

Since Alzheimer’s (APP/PS1) and TD2 (db/db) pathologies cooperated to increase expression of diverse cytokines beyond either pathology alone (Fig. 2), we next asked if prediabetic conditions would have a similar effect. To test this, we exposed APP/PS1 mice to high-fat diet (HFD, see the “Materials and methods” section) for 5–6 months. We then quantified the same panel of 22 cytokines from mouse cortical tissues (Fig. 3, Additional file 1: Figure S6). Using PLSDA analysis to identify a profile of cytokines most upregulated in response to APP/PS1 and HFD pathology, we observed that HFD combined with APP/PS1 pathology elevated a profile of cytokines compared to wild-type controls (Additional file 1: Figure S6B-C).

Again, we used PLSDA to evaluate the differences between combined APP/PS1-HFD pathology and either APP/PS1 or HFD alone (Fig. 3b). Similar to our findings with db/db mice, we identified an LV1 that separated APP/PS1-HFD from both HFD and APP/PS1 groups (Fig. 3c, d) and a second profile, LV2, that separated HFD only from both other groups (Fig. 3e, f). LV1 consisted of a weighted combination of cytokines that were particularly elevated in response to combined APP/PS1-HFD. Importantly, the top correlates with APP/PS1-HFD on LV1 included MIP-1α, IL-1β, eotaxin, and IL-17, reflecting the chemotactic and pro-inflammatory properties of cytokines found in the combined models in Figs. 1 and 2. We also found that top cytokines from LV1 followed a similar trend to the samples scored on LV1 (Fig. 3d and Additional file 1: Figure S7).

### Plasma Aβ correlates with brain cytokines in APP/PS1xdb/db mice

**Fig. 4:**
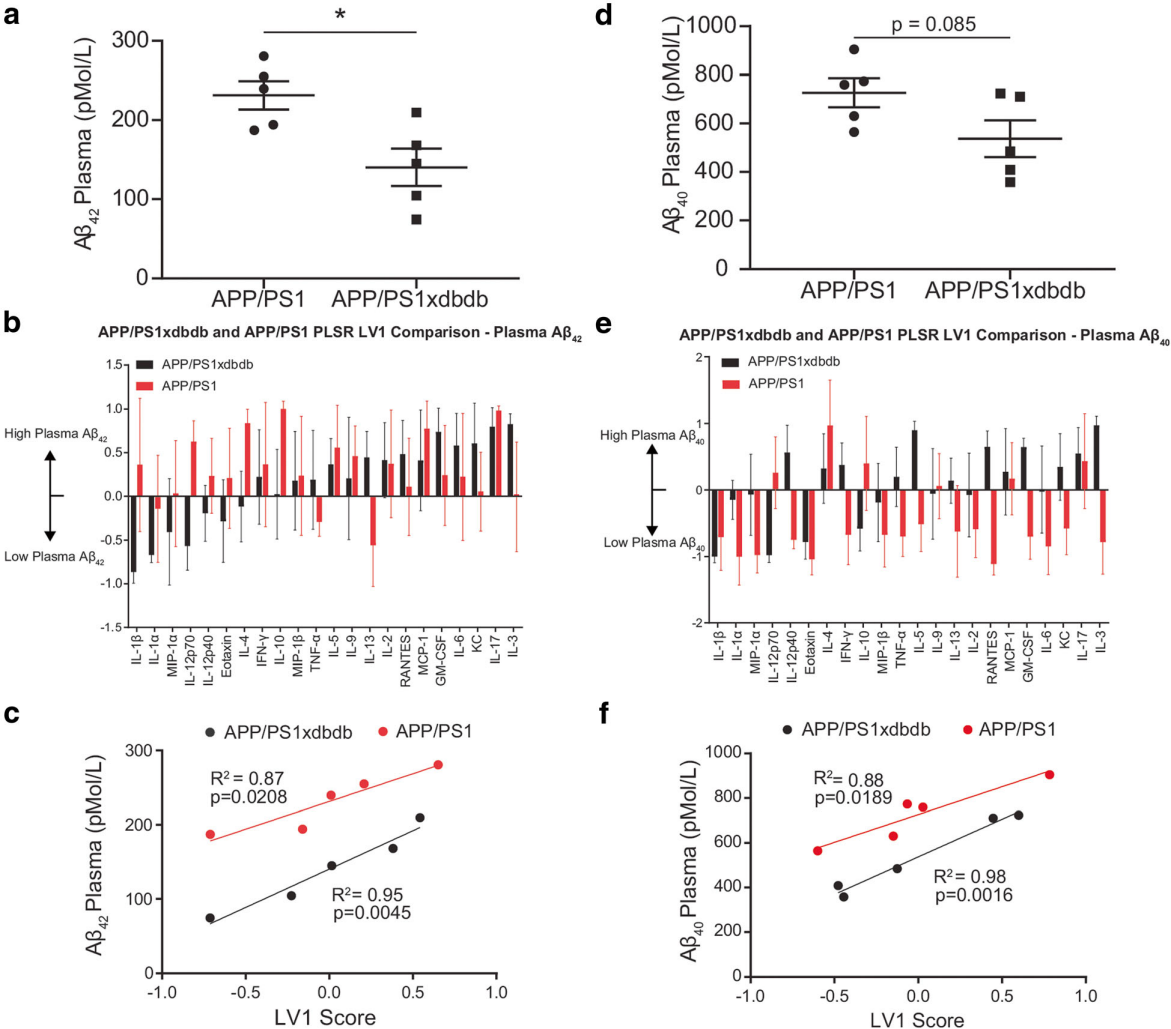
Plasma amyloid levels correlated with distinct signatures of brain cytokine expression in APP/PS1 or APP/PS1xdb/db mice. **a** Plasma Aβ1-42 levels were significantly decreased in APP/PS1xdb/db compared to APP/PS1 mice (mean ± SEM; *p < 0.05, Student’s *t* test). **b** Distinct profiles of cytokines correlated with Aβ1-42 levels in db/db or APP/PS1xdb/db mice (mean ± SD in an iterative subsampling of 80% of samples). **c** Plasma Aβ1-42 levels were decreased in APP/PS1xdb/db compared to APP/PS1 mice and significantly correlated with brain composite cytokine score on LV1. **d** Plasma Aβ1-40 levels trend towards a decrease in APP/PS1xdb/db compared to APP/PS1 mice (mean ± SEM; *p* = 0.085, Student’s *t* test) **e** Distinct profiles of cytokines correlated with Aβ1-40 levels in db/db or APP/PS1xdb/db mice, ordered with respect to **b** (mean ± SD in an iterative subsampling of 80% of samples). **f** Plasma Aβ1-40 concentration was decreased in APP/PS1xdb/db compared to controls significantly correlated with brain composite cytokine score on LV1. Data were collected from ten animals (4 M/6F, APP/PS1 1 M/4F, APP/PS1xdb/db 3 M/2F)

Given that T2D diabetes, as modeled by db/db and HFD mice, amplified brain Aβ levels (Additional file 1: Figure S1B) and enhanced pro-inflammatory cytokine production (Figs. 2 and 3), we next hypothesized that peripheral plasma levels would correlate with brain cytokine expression in APP/PS1xdb/db mice. We found that plasma Aβ levels quantified from blood collected at euthanasia were lower in APP/PS1xdb/db mice (Aβ40 *p = 0.014 vs. APP/PS1; Aβ40 *p* = 0.085) (Figs. 4a, d). To identify a relationship between Aβ and cytokines, we used PLSR analysis to regress brain tissue cytokine measurements against plasma measurements of Aβ1-40 or Aβ1-42 from the same animals (Fig. 4). In APP/PS1 mice, we found that elevated plasma Aβ1-42 was correlated with increased expression of a number of anti-inflammatory cytokines, including IL-10 and IL-4 (Figs. 4b, c). In contrast, high plasma Aβ1-42 correlated with primarily pro-inflammatory cytokines in APP/PS1xdb/db mice, including IL-3, IL-17, and KC (CXCL1). In terms of Aβ1-40, PLSR analysis revealed that increased plasma levels were associated with increased IL-4 and broad suppression of pro-inflammatory cytokines whereas pro-inflammatory cytokines were elevated in APP/PS1xdb/db mice with low plasma levels (Figs. 4e, f). These data suggest a complex relationship between neuroinflammation and pathology in line with previous observations in APP/PS1xdb/db mice in which the overall inflammation is exacerbated in SP-free areas [17].

### Glucose and insulin correlate with brain cytokines in APP/PS1xdb/db mice

**Fig. 5:**
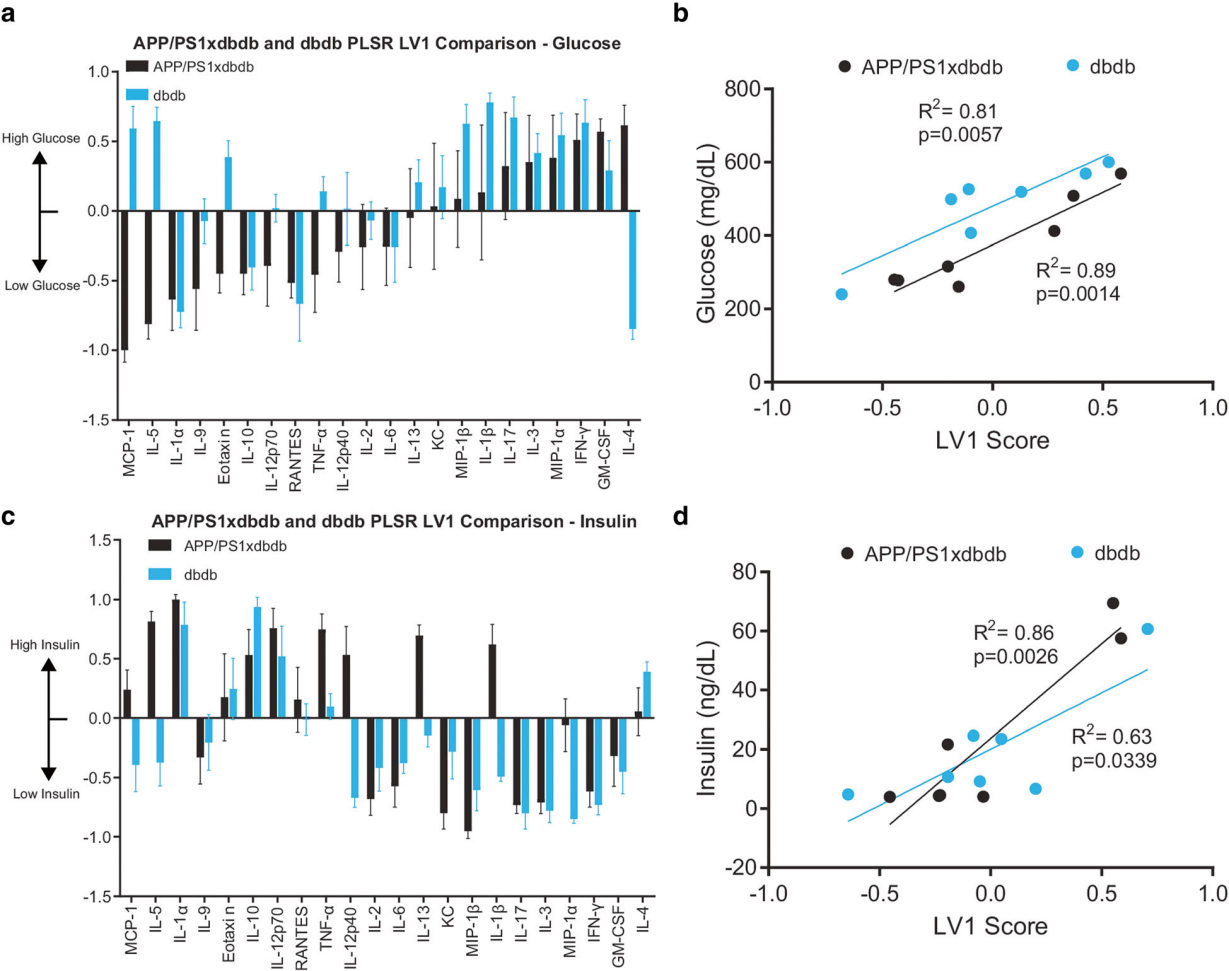
Plasma glucose and insulin levels correlate with brain cytokine expression in db/db and mixed models. **a** Profiles of cytokines correlated with glucose levels in db/db or APP/PS1xdb/db mice (mean ± SD in an iterative subsampling of 80% of samples). **b** Glucose levels significantly correlate with brain composite cytokine score on LV1. **c** Profiles of cytokines correlated with insulin levels in db/db or APP/PS1xdb/db mice ordered with respect to **a** (mean ± SD in an iterative subsampling of 80% of samples). **d** Plasma insulin concentration significantly correlated with brain composite cytokine score on LV1. Data were collected from 14 animals (8 M/6F, db/db 2 M/5F, APP/PS1xdb/db 6 M/1F)

T2D models stimulate Aβ pathology and cytokine expression (Figs. 2 and 3). Since T2D drives dysregulation of glucose and insulin, we concluded this study by asking if these variables correlated with brain cytokine levels. While we found that high glucose strongly correlated with brain cytokines in db/db mice, we found that high glucose most strongly correlated with elevated anti-inflammatory IL-4 in APP/PS1xdb/db mice (Fig. 5a, b). In contrast, low insulin strongly correlated with a strongly pro-inflammatory signature, including MIP-1β, KC (CXCL1), and IL-13 in both db/db and APP/PS1xdb/db mice (Fig. 5c, d). These findings indicate that neuroinflammation is tightly linked to glucose and insulin levels, even in the db/db genetic mouse model.

## Discussion

The close relationship between diabetes and AD has been long explored, both in epidemiological studies [5, 6] and murine models [11, 17]. Although the underlying mechanisms by which diabetes promotes AD pathogenesis have not been elucidated, previous research supports multi-faceted dysfunction caused by diabetes, including neuronal insulin signaling, mitochondrial dysfunction, and inflammation [42, 43]. Inflammation is a relevant feature of AD and diabetes and it plays critical roles in the pathogenesis of both diseases [43]. To our knowledge, this is the first study to holistically analyze complex cytokine profiles in mixed models harboring prediabetes, T1D, or T2D together with amyloid pathology relevant to AD. Our analysis revealed that diabetic or prediabetic pathologies cooperatively modulated expression of pro-inflammatory cytokines in the brains of APP/PS1 mice and showed that profiles of expressed cytokines strongly correlated with circulating glucose levels.

Our study employed classical models of prediabetes and T1D in APP/PS1 mice. As previously described, HFD-induced prediabetes leads to severe hyperinsulinemia that modulates glucose levels, while STZ administration results in a well-characterized model of T1D with low insulin and high glucose levels. We also included a newer mixed animal model of AD-T2D, by crossing APP/PS1 with db/db mice [17, 31]. These mice are severely overweight, and they present insulin resistance. In this context, high insulin levels fail to control hyperglycemia. Within the brain, APP/PS1xdb/db mice showed increased tau phosphorylation that initially affects the cortex and spreads to the hippocampus [17, 31]. We also previously observed that APP/PS1xdb/db mice exhibit a shift in the kinetics of Aβ deposition, and while more toxic soluble Aβ species are increased, insoluble Aβ and senile plaques are reduced [17, 31]. In APP/PS1xdb/db mice, we also observed that plasma Aβ levels were reduced when compared with APP/PS1 mice. Although still controversial, our data are in line with the “peripheral sink” hypothesis of Aβ [44–46]. In this context, reduced plasma Aβ in APP/PS1xdb/db mice supports the observed increase in brain soluble Aβ levels. Other studies using similar mouse models have also detected changes in Aβ. In particular, Niedowicz et al. [47] did not detect significant changes in cortical Aβ deposition; however, the combination of AD and T2D increased oligomeric Aβ in the brain. Also, while total Aβ levels are not altered in young APP23xob/ob mice, these mice have been found to present an increase in amyloid angiopathy [48]. Because of reported pathological differences between combined AD/diabetic models, our present study includes models of three different metabolic alterations relevant to diabetes, enabling us to make robust conclusions about the chronic effects of diabetes on cytokine expression in the AD brain.

While cytokines have traditionally been divided into pro- and anti-inflammatory categories, they are often pleiotropic, and cytokines with opposing functions are often expressed together. Moreover, the complexity of the neuroinflammatory response may be magnified when multiple chronic inflammatory processes present together, as with APP/PS1xdb/db mice. These complex alterations necessitate simultaneous measurement and analysis of a panel of cytokines to understand the effects of diabetic pathology on neuroinflammation in APP/PS1 AD mice. As our group has previously shown, PLSR analysis provides a powerful tool to detect changes in cytokine expression associated within a pathological group or with measures of pathological severity. Moreover, the *profiles* of cytokines generated on each LV provide a ranking of the relative importance of each cytokine in distinguishing among groups, enabling us to identify the functions of top cytokines involved in each separation. In the present study, we used this same (PLSDA) approach to identify profiles of cytokines that were most different between single AD or diabetic pathologies, and in combined APP/PS1xdiabetic or APP/PS1xprediabetic mice. Regardless of the diabetic insult (HFD, STZ, db/db), our data revealed that diabetic conditions cooperated with APP/PS1 pathology to strongly upregulate cytokine expression in the combined model. We note that there were few differences between 6-month-old wild-type and APP/PS1 mice, due to this being an early pathological time point in this model [29], while prediabetes or diabetes animal models produce broad inflammation at earlier time points [49]. Therefore, the severity of combined diabetic and APP/PS1 pathology precludes studying the effects of advanced age using these models.

Since the goal of the present study was to determine the effects of combined AD-diabetic pathology, we conducted our analyses comparing each diabetic model to APP/PS1 mice in the absence of diabetic pathology. The LV1 cytokine profile for each of these models significantly separated combined APP/PS1 and diabetic/prediabetic mice from either pathology alone. The top cytokines associated with combined APP/PS1 and diabetic/prediabetic pathology in all three metabolic models had pro-inflammatory and chemotactic properties (e.g., MIP-1β, MIP-1α, MCP-1, IFN-γ) [50–53]. Top correlates in each LV1 identified chemokines (MCP-1 in APP/PS1-STZ, MIP-1α and MIP-1β in APP/PS1xdb/db, MIP-1α in APP/PS1-HFD) that were only significantly upregulated in the presence of combined pathology (Figs. 1, 2, and 3, Additional file 1: Figures S3, S5, S7), emphasizing that the combined presence of amyloid and metabolic pathologies cooperatively modulates the neuroinflammatory environment.

We also used PLSR analysis to identify profiles of brain cytokines that were strongly correlated with increased plasma glucose or insulin in our mixed APP/PS1xdb/db (AD-T2D) model. Cytokines, as inflammatory markers, have been previously analyzed in diabetic patients [54, 55] and diabetes animal models [56, 57]. Our PLSR-based profiling analysis confirmed some previous observations on individual cytokines. First, the strongest glucose-cytokine signals identified by the model included granulocyte-macrophage colony-stimulating factor (GM-CSF), IFN-γ, and IL-3, all of which appeared within the top six correlates for LV1 based on the PLSDA analysis (Fig. 2). Of these, GM-CSF promotes inflammation in various infectious and inflammatory diseases, and it is implicated in monocyte/macrophage activation [58]. Previous studies of diabetes patients have reported that circulating GM-CSF levels are not affected [59]; however, it has also been shown that GM-CSF levels are increased in diabetes [55, 60, 61] as well as in prediabetic patients, correlating with glycosylated hemoglobin [62]. Likewise, studies in T2D patients show that insulin and oral hypoglycemic agents may reduce serum GM-CSF levels in T2D patients [61]. Additionally, intracerebral GM-CSF administration to APP/PS1 mice directly increases blood-brain barrier endothelial permeability, suggesting that high levels of GM-CSF detected in the brain parenchyma and CSF of AD patients may induce blood-brain barrier opening. Moreover, GM-CSF blockade abolishes monocyte infiltration in the brain from APP/PS1 mice [58]. Also, GM-CSF administration in a phase Ib/II clinical trial on renal cell carcinoma has been associated with acute multifocal cerebral venous thrombosis and subdural and subarachnoid hemorrhage [63]. Since diabetes also affects vascular integrity and blood-brain barrier [64], it is feasible that the increased spontaneous central bleeding observed in APP/PS1xdb/db mice [17, 31] is related, at least in part, to GM-CSF-glucose association.

PLSR analysis revealed that IFN-γ was also a top correlate with high glucose levels in AD-T2D mice because it has been previously identified as an inflammatory mediator in AD [65]. IFN-γ plasma levels correlated with glycosylated hemoglobin, which is a biomarker of average glucose levels, in prediabetic patients [62]. Higher levels of IFN-γ were also detected in T2D patients [66], leading to beta-cell dysfunction. Moreover, IFN-γ may play a role in the genesis of insulin resistance [67]. Also, abnormally high levels of IFN-γ protein are detected in brain and blood serum of diabetic mice, and blocking IFN-γ has been shown to restore microglial chemotactic response to vascular damage [68]. Intracerebral hemorrhage is also associated with high levels of IFN-γ [69, 70]. Blood-brain barrier alterations and chronic inflammation are classical pathological features of cerebral small vessel disease, characterized by multiple strokes, blood-brain barrier dysfunction, and chronic inflammation at the neurovascular unit [71]. In line with these observations, db/db [72] and APP/PS1xdb/db mice [17, 31] show widespread spontaneous bleeding. In this context, IFN-γ may lead to diffuse neuron and oligodendrocyte damage [71].

IL-17 was also upregulated in all three diabetic models and was highly correlated with peripheral Aβ1-42 and with glucose levels in db/db mice. IL-17 is the most effective cytokine of T helper 17 cells and plays a pro-inflammatory role in chronic inflammation [73] observed in T2D [74]. Also, IL-17 production has been associated with cerebral small vessel disease, similar to that observed in db/db and APP/PS1xdb/db mice [75], and IL-17 might contribute to atherosclerosis development. Moreover, IL-17 has been implicated in the neuroinflammatory response in AD [76], and while some controversial studies show a protective role for IL-17 against the risk for T2D [77], others support a crucial role for IL-17 in inflammation, insulin resistance, and T2D [66, 78]. In line with these studies, IL-17 levels are also increased in the hippocampus from db/db mice [73], and antibodies targeting Th17 cells have been studied in an effort, to protect individuals at risk for developing diabetes [79]. These data support that metabolic alterations can broadly trigger and exacerbate brain neuroinflammation and production of cytokines known to promote T2D and AD pathogenesis [36, 79].

PLSR analysis also revealed changes in cytokine expression associated with high insulin levels and insulin resistance in our mixed AD-T2D model, and both insulin and insulin resistance are major contributors to central complications in AD and T2D [4]. IL1-α, IL-5, IL-12p70, tumor necrosis factor (TNF-α), and IL1-β are highly correlated with insulin levels in APP/PS1xdb/db mice. IL-1 family of cytokines plays a relevant role in the response to inflammatory stress, in close association with T2D. Previous studies have shown that insulin favors a pro-inflammatory state via insulin receptor, glucose metabolism, production of reactive oxygen species, and secretion of IL-1 [80]. Also, pancreatic β-cell IL-1 expression is increased in T2D patients [81]. In this sense, IL1-α and β blockage show an improvement in insulin secretion and glycaemia [82]. In line with these observations, IL-1β has been reported to lead to the reduction of insulin-induced glucose uptake and insulin resistance [83]. In short-term studies with mice on high-fat diet, serum IL-1α and IL-1β do not seem to be affected [84]. However, longer exposure to HFD and insulin resistance increases IL-1*β* mRNA in the hippocampus [85]. IL-1β has also been proposed as a contributor to the onset of AD [86]. Likewise, studies in non-obese diabetic mice have reported that IL-1α is increased in plasma and insulin therapy increases IL-1α release in splenocytes [87]. Moreover, IL-1 usually synergizes with TNF-α, since both cytokines are produced at sites of local inflammation [81]. On the other hand, TNF-α has been shown to cause cellular insulin resistance in hypothalamic neurons [88]. Also, prediabetes with high insulin levels appears to increase TNF-α in patients [62]. Similar outcomes have been observed in prediabetic mice, in which long-term exposure to high-fat diet and insulin resistance increases TNF*-α* protein in the hippocampus [85]. Moreover, increased levels of TNF-α are detected in diabetic patients and may serve as a prognostic tool for diabetic retinopathy [78]. Central administration of Aβ oligomers induces peripheral glucose intolerance. However, this effect is avoided in TNF-α receptor 1 knockout mice, supporting a role for TNF-α in the two-way crosstalk between AD and diabetes [89].

The second top correlate with insulin in APP/PS1xdb/db mice was IL-5. Certain studies have found IL-5 to be reduced in diabetes [78], while others have found IL-5 plasma levels to be correlated with glycosylated hemoglobin in diabetic patients [62]. Similarly, increased levels of IL-5 have been shown in HFD-fed mice [90]. We also found IL-12p70 and IL-13 to be closely related to high insulin levels in APP/PS1xdb/db mice, both of which have been shown to be elevated in prediabetic patients [62]. A similar trend has been observed for IL-12p70 in HFD-fed mice [91]. Altogether, multivariate analysis of cytokine expression in the cortex from our mixed model indicates that there are marked pro-inflammatory differences in cytokine profiles associated with the co-presentation of T2D and AD pathologies.

Our findings in the current study motivate a number of future avenues of research. First, although we have found that diabetic pathology robustly increased cytokine expression in cortical tissues, with or without amyloid pathology, we have not identified the cell type expressing each one. Given that metabolic dysregulation particularly affects neurons, it is possible that neurons contribute to cytokine expression, as we have recently found in the context of brain injury [35]. Although we note that neuroinflammatory response is also mediated by astrocytes, prior studies in our lab [31] have revealed limited differences in astrocyte burden in APP/PS1xdb/db compared to APP/PS1 animals. Nevertheless, more detailed future astroglial studies should be carried out in different metabolic disease-AD models. Second, given that cytokine expression is regulated by intracellular phospho-signaling pathways, it is likely we will identify dysregulation of central signaling pathways, such as PI3K/Akt, NFκB, or MAPK, that may be targeted using small molecules to modulate neuroinflammation. Finally, it remains unknown if metabolism normalizing therapies, such as insulin, have the potential to reduce the neuroinflammatory signatures identified here.

## Conclusions

In total, our multiplexed analysis of cytokines shows that Alzheimer’s and diabetic pathologies cooperate to enhance profiles of cytokines reported to be involved in both diseases. Our analysis identified pro-inflammatory cytokines that were upregulated in prediabetic, T2D diabetic, and T1D diabetic models. Therefore, these data suggest that metabolic dysregulation drives neuroinflammation, regardless of the underlying cause.

## Supplementary information

**Additional file 1: Figure S1.** Metabolic assessment, amyloid pathology and microglia burden are altered in mixed models of metabolic disease and AD. **Figure S2.** APP/PS1-STZ pathology upregulates a profile of cytokines compared to wild-type control. **Figure S3.** Individual cytokines measured for APP/PS1, STZ, and APP/PS1-STZ groups. **Figure S4.** APP/PS1xdbdb pathology upregulates a profile of cytokines compared to wild-type controls. **Figure S5.** Individual cytokines measured for APP/PS1, dbdb, and APP/PS1xdbdb groups. **Figure S6.** APP/PS1-HFD pathology upregulates a profile of cytokines compared to wild-type controls. **Figure S7.** Individual cytokines measured for APP/PS1, HFD, and APP/PS1-HFD groups.

## Data Availability

Data is available upon reasonable request.
