# Supplementary material for: Amyloid beta and diabetic pathology cooperatively stimulate cytokine expression in an Alzheimer’s mouse model
Source: J Neuroinflammation. 2020 Jan 28;17:38. doi: 10.1186/s12974-020-1707-x (PMC6988295; doi:10.1186/s12974-020-1707-x)
Supplement: Supplementary file 1 — Additional file 1: Figure S1. Metabolic assessment, amyloid pathology and microglia burden are altered in mixed models of metabolic disease and AD. Figure S2. APP/PS1-STZ pathology upregulates a profile of cytokines compared to wild-type control. Figure S3. Individual cytokines measured for APP/PS1, STZ, and APP/PS1-STZ groups. Figure S4. APP/PS1xdbdb pathology upregulates a profile of cytokines compared to wild-type controls. Figure S5. Individual cytokines measured for APP/PS1, dbdb, and APP/PS1xdbdb groups. Figure S6. APP/PS1-HFD pathology upregulates a profile of cytokines compared to wild-type controls. Figure S7. Individual cytokines measured for APP/PS1, HFD, and APP/PS1-HFD groups. [file 12974_2020_1707_MOESM1_ESM.docx]

**Amyloid-beta and diabetic pathology cooperatively stimulate cytokine expression in an Alzheimer’s mouse model**

Sitara B. Sankar^1^, Carmen Infante-Garcia^2,3^, Laura D. Weinstock^1^, Juan Jose Ramos-Rodriguez^2,4^, Carmen Hierro-Bujalance^2,3^, Cecilia Fernandez-Ponce^3,5^, Levi B. Wood^1,6,¥,^*, Monica Garcia-Alloza^2,¥,^*

In memoriam of Maria Isabel Murillo-Carretero

^1^Wallace H. Coulter Department of Biomedical Engineering, Georgia Institute of Technology, Atlanta, GA 30332 USA

^2^Division of Physiology. School of Medicine, Universidad de Cadiz. Cadiz, Spain.

^3^Instituto de Investigacion Biomedica de Cadiz (INIBICA), Cadiz.

^4^Currently at Department of Physiology, School of Health Sciences, Granada University, Spain.

^5^Division of Immunology, School of Medicine, Universidad de Cadiz. Cadiz, Spain.

^6^George W. Woodruff School of Mechanical Engineering and Parker H. Petit Institute for Bioengineering & Bioscience, Georgia Institute of Technology, Atlanta, GA 30332 USA

**Running title:** Diabetic conditions stimulate expression of cytokines in Alzheimer’s mice.

^¥^Equally contributing senior authors

*Address correspondence to:

| Levi B. Wood  George W. Woodruff School of Mechanical Engineering  Parker H. Petit Institute for Bioengineering & Bioscience  Georgia Institute of Technology  315 Ferst Dr, Rm 3303  Atlanta, GA 30332  [levi.wood@me.gatech.edu](mailto:levi.wood@me.gatech.edu) | Monica Garcia-Alloza  Division of Physiology.School of Medicine.  Universidad de Cadiz. Instituto de Investigacion Biomedica de Cadiz (INIBICA).  Cadiz, Spain.  [monica.garcia@uca.es](mailto:monica.garcia@uca.es) |
| --- | --- |


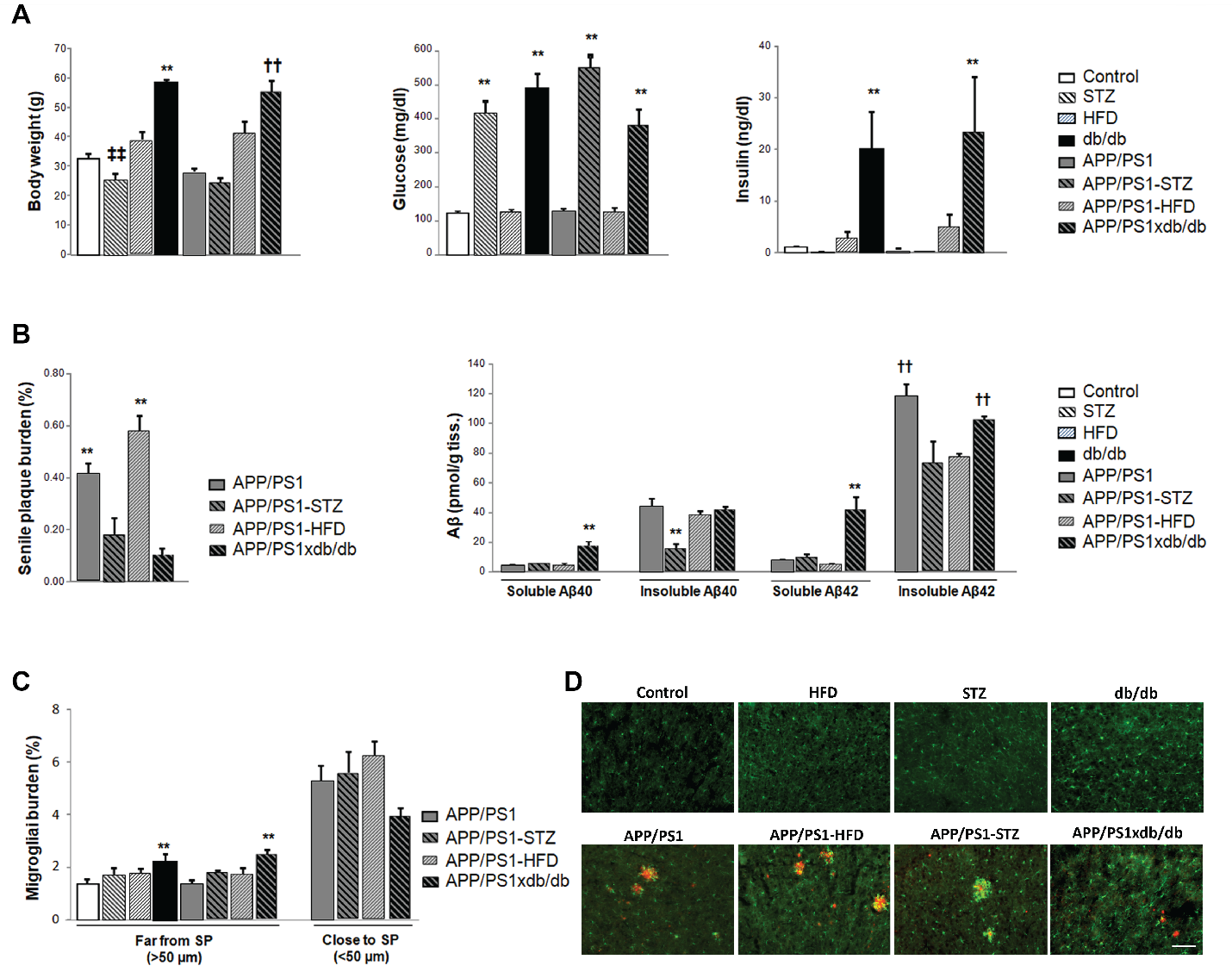


**Figure S1. Metabolic assessment, amyloid pathology and microglia burden are altered in mixed models of metabolic disease and AD. A.** Body weight was significantly increased in mice on HFD, and this effect was more severe in diabetic animals (db/db and APP/PS1xdb/db) (**p<0.01 vs. Control, HFD, STZ, APP/PS1, APP/PS1-HFD and APP/PS1-STZ; ††p<0.01 vs. Control, STZ, APP/PS1 and APP/PS1-STZ; ‡‡p<0.01 vs. Control and HFD). Glucose levels were significantly increased after inducing T1D by STZ treatment, both in control and APP/PS1 mice, as well as in T2D mice with and without AD (db/db and APP/PS1xdb/db mice) (**p<0.01 vs. Control, HFD, APP/PS1 and APP/PS1-HFD). A slight increase in insulin levels was observed in HFD mice, however differences only reached statistical significance in T2D mice (db/db and APP/PS1xdb/db) (**p<0.01 vs. Control, HFD, STZ, APP/PS1, APP/PS1-HFD and APP/PS1-STZ). Data are presented as mean ±SEM. Control n=14 (10 male and 4 female), HFD n=7 (4 male and 3 female), STZ n=7 (5 male and 2 female), db/db n=7 (2 male and 5 female), APP/PS1 n=14 (5 male and 9 female), APP/PS1-HFD n=7 (4 male and 3 female), APP/PS1-STZ n=7 (4 male and 3 female), APP/PS1xdb/db n=7 (4 male and 3 female). Differences detected by one-way ANOVA followed by Tuckey b or Tamhane tests. **B.** Amyloid pathology was changed in mice with metabolic alterations. SP burden was significantly lower in APP/PS1-STZ and APP/PS1xdb/db mice (**p<0.01 vs. APP/PS1-STZ and APP/PS1xdb/db). Soluble Aβ levels are increased in APP/PS1xdb/db mice (Aβ40: **p<0.01 vs. rest of the groups; Aβ42: **p<0.01 vs. rest of the groups). Insoluble Aβ40 levels were lower in APP/PS1-STZ mice (Aβ40: **p<0.01 vs. rest of the groups; Aβ42: ††p<0.01 vs. APP/PS1-HFD). Data are presented as mean ±SEM. Control n=10 (7 male and 3 female), HFD n=5 (3 male and 3 female), STZ n=5 (3 male and 2 female), db/db n=5 (1 male and 4 female), APP/PS1 n=5 (3 male and 7 female), APP/PS1-HFD n=5 (3 male and 2 female), APP/PS1-STZ n=5 (3 male and 2 female), APP/PS1xdb/db n=5 (3 male and 7 female) and 6 sections/mouse were included in the study. Differences detected by one-way ANOVA followed by Tuckey b or Tamhane tests. **C.** Microglia burden. Microglia burden was increased in SP-free areas in db/db and APP/PS1xdb/db mice when all groups under study were compared ([F_(7,42)=_4.68, **p=0.001 vs. Control and APP/PS1]). Differences did not reach statistical significance in close proximity to plaques when SP-bearing groups were compared ([F_(7,21)_=1.98, p=0.147]). Data are presented as mean ±SEM. Control n=10 (7 male and 3 female), HFD n=5 (3 male and 3 female), STZ n=5 (3 male and 2 female), db/db n=5 (1 male and 4 female), APP/PS1 n=5 (3 male and 3 female), APP/PS1-HFD n=5 (3 male and 2 female), APP/PS1-STZ n=5 (3 male and 2 female), APP/PS1xdb/db n=5 (3 male and 3 female) and 6 sections/mouse were included in the study. Differences detected by one-way ANOVA followed by Tuckey b test. **D.** Illustrative example of 4G8 (Aβ, red) and Iba1 (microglia, green) immunostaining in a cortical section from all groups under study. Scale bar= 100 µm.


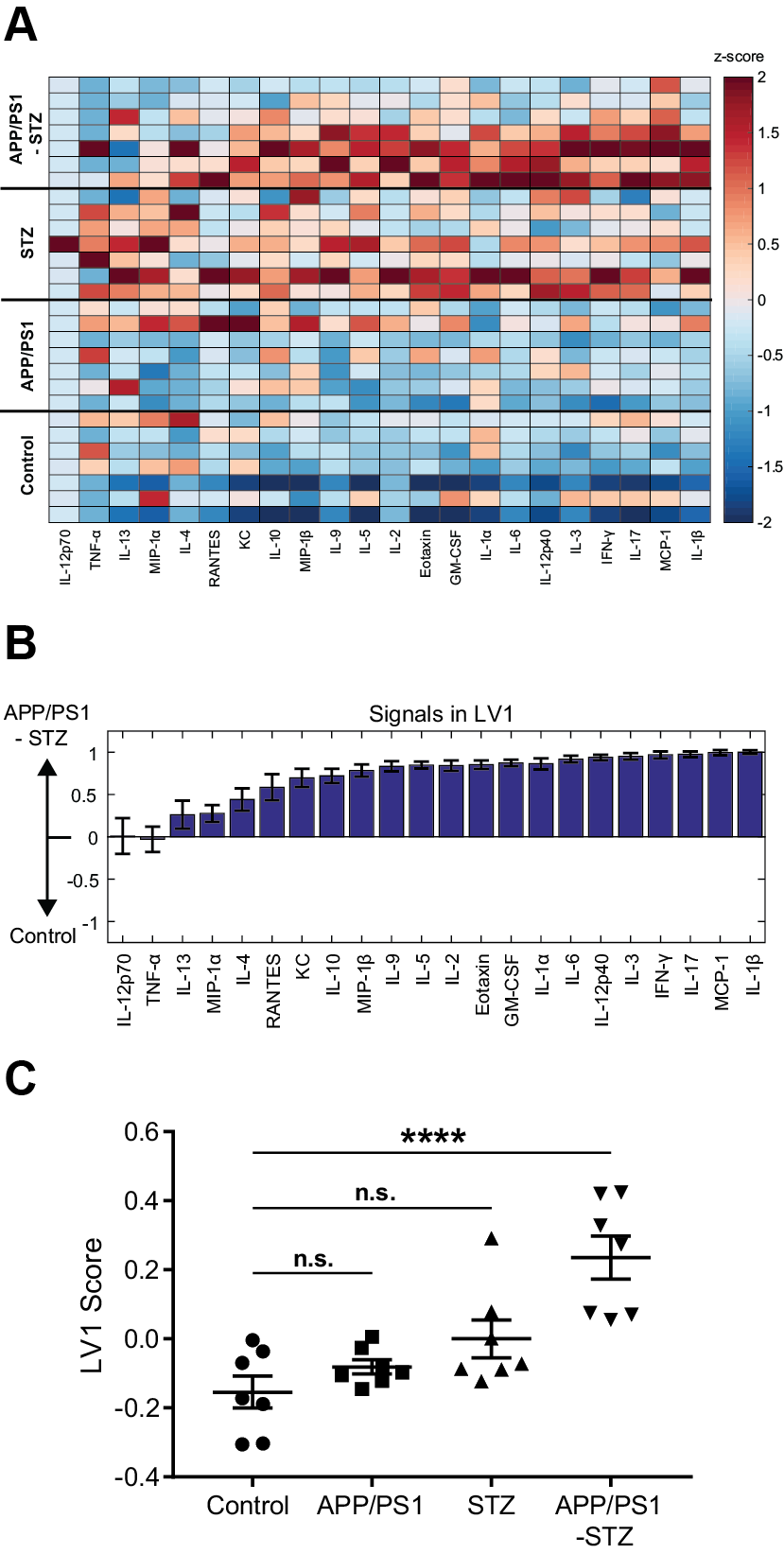


**Figure S2. APP/PS1-STZ pathology upregulates a profile of cytokines compared to wild-type control.**

**A.** Luminex analysis of 22 cytokines (columns, z-scored) expressed in the cortex of control (wild-type), APP/PS1, STZ, and APP/PS1-STZ mice (each row is a cortex sample). **B.** Discriminant partial least squares (PLSDA) analysis identified a profile of cytokines, LV1, that best distinguished APP/PS1-STZ mice (positive) from controls (negative). Errors bars on each cytokine were computed by PLSDA model regeneration using iterative subsampling of 80% of the samples (mean ± SD). **C.** Scoring the data for each sample in A on LV1 revealed that APP/PS1-STZ group is significantly increased on the LV1 cytokine profile compared to control (*****p*<0.0001; ordinary one-way ANOVA with Dunnett’s test).

**
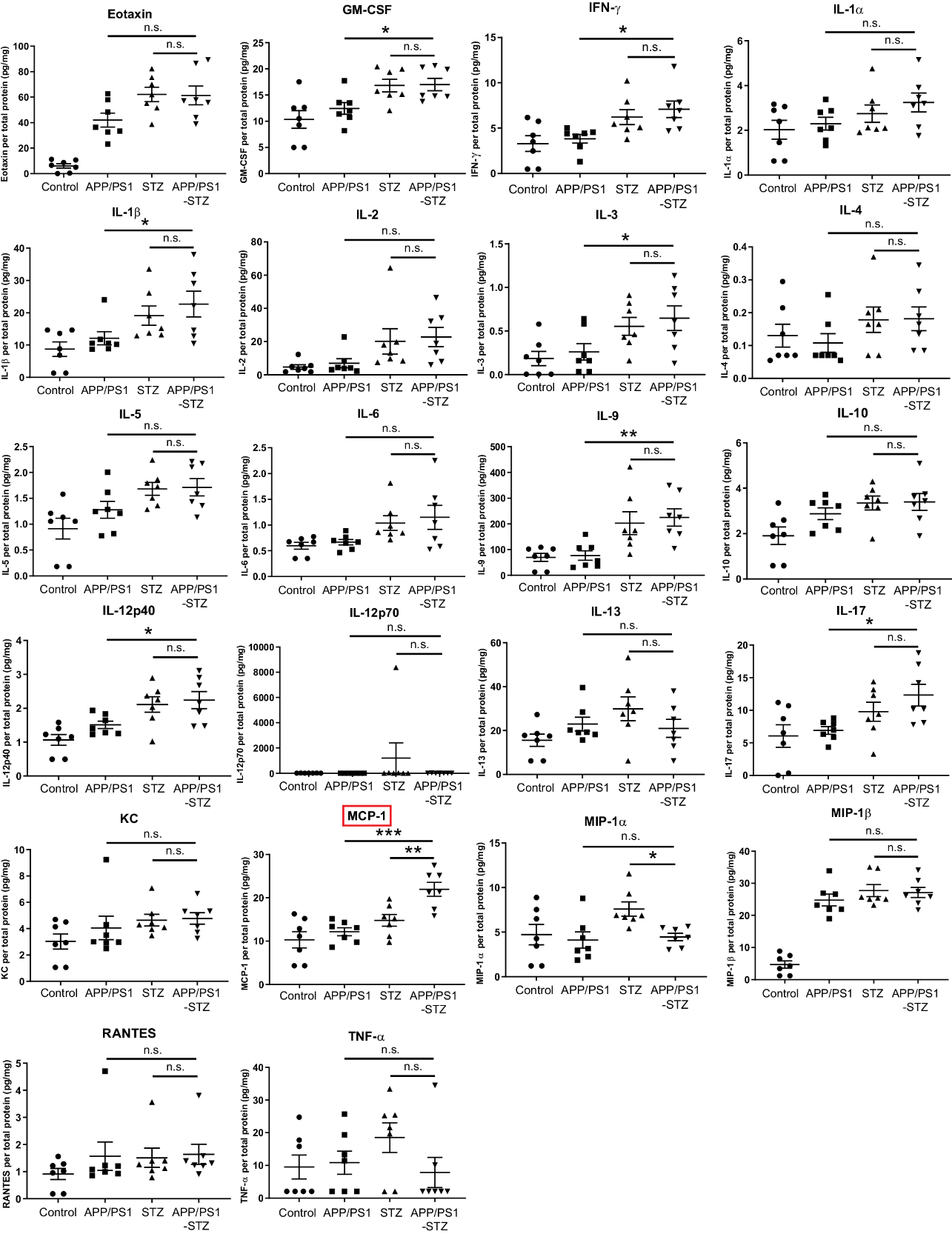
**

**Figure S3. Individual cytokines measured for APP/PS1, STZ, and APP/PS1-STZ groups.**

Bar plots of 22 analytes measured for APP/PS1, STZ, and APP/PS1-STZ groups. APP/PS1-STZ pathology upregulates GM-CSF, IFN-γ, IL-9, IL-12p40, IL-17, and MCP-1 when compared to APP/PS1, and MCP-1 only when compared to STZ (**p<*0.05, ***p<*0.01, ****p<*0.001; ordinary one-way ANOVA among APP/PS1, STZ, and APP/PS1-STZ groups with Holm-Sidak’s test). Red box highlights cytokine that is significantly up-regulated in combined group compared to both APP/PS1 and STZ models.

**
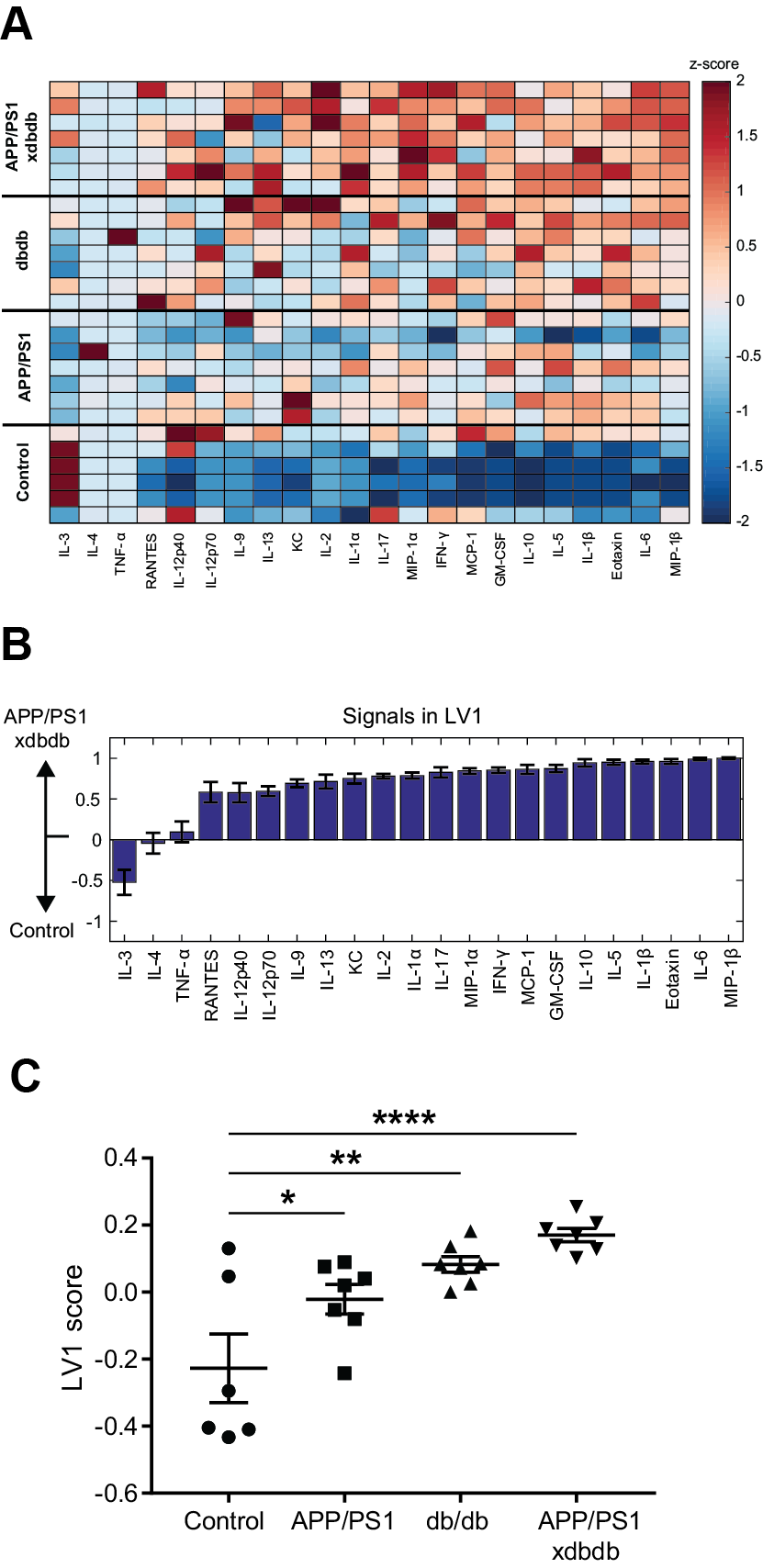
**

**Figure S4. APP/PS1xdbdb pathology upregulates a profile of cytokines compared to wild-type controls.**

**A.** Luminex analysis of 22 cytokines (columns, z-scored) expressed in the cortex of control (wild-type), APP/PS1, dbdb, and APP/PS1xdbdb mice (each row is a cortex sample). **B.** PLSDA identified a profile of cytokines, LV1, that best distinguished APP/PS1xdbdb mice (positive) from controls (negative). Errors bars on each cytokine were computed by PLSDA model regeneration using iterative subsampling of 80% of the samples (mean ± SD). **C.** Scoring the data for each sample in **A** on LV1 revealed that the APP/PS1, dbdb, and APP/PS1xdbdb groups are increased on the LV1 cytokine profile compared to control (**p<*0.05, ***p<*0.01,*****p*<0.0001; ordinary one-way ANOVA with Dunnett’s test).

**
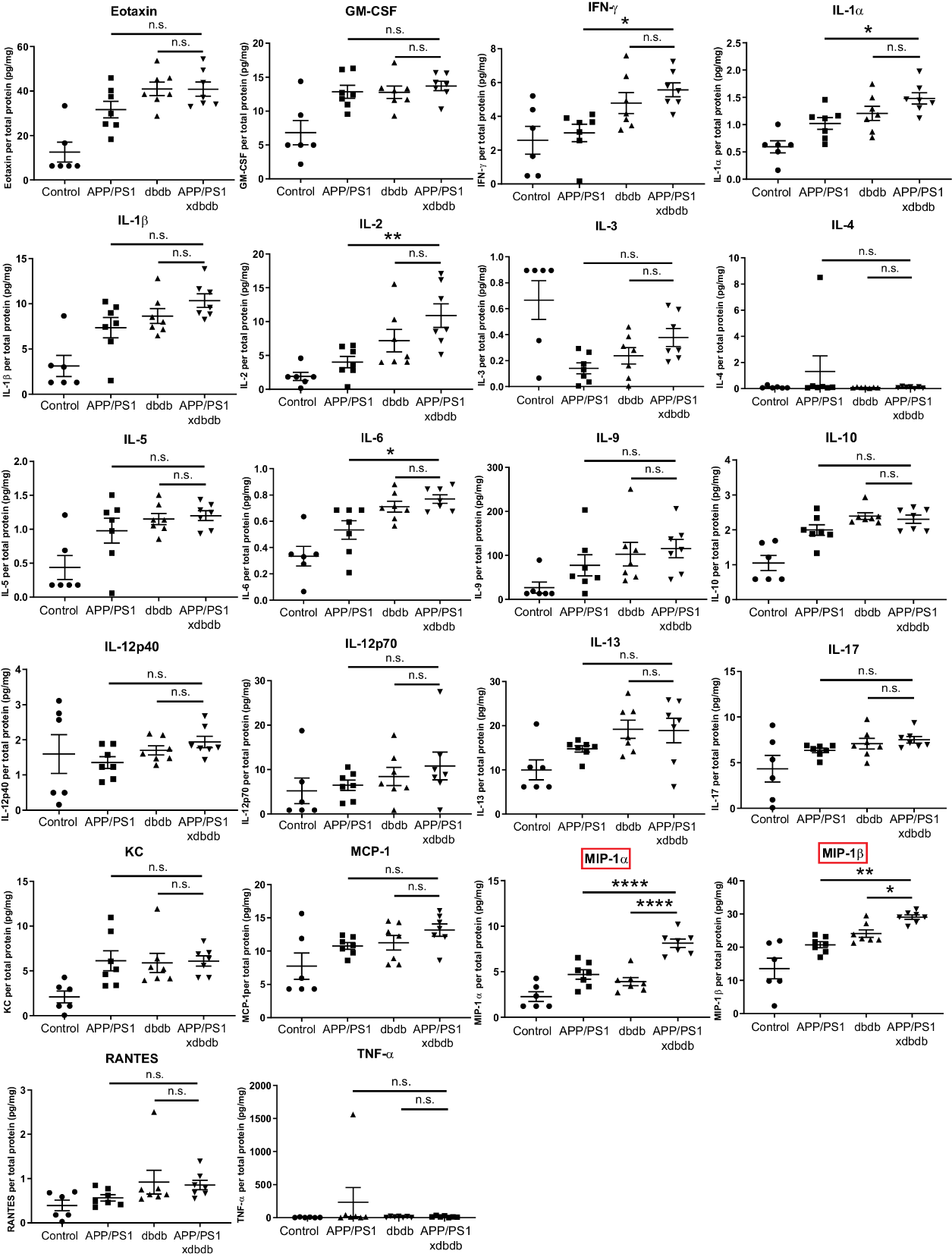
**

**Figure S5. Individual cytokines measured for APP/PS1, dbdb, and APP/PS1xdbdb groups.**

Bar plots of 22 analytes measured for APP/PS1, STZ, and APP/PS1-STZ groups. APP/PS1-STZ pathology upregulates IFN-γ, IL-1α, IL-2, IL-3, IL-6, IL-12p40, IL-17, MIP-1α, and MIP-1β when compared to APP/PS1, and MIP-1α and MIP-1β only when compared to dbdb (**p<*0.05, ***p<*0.01, *****p<*0.0001; ordinary one-way ANOVA among APP/PS1, dbdb, and APP/PS1xdbdb groups with Holm-Sidak’s test). Red boxes highlight cytokines that are significantly up-regulated in combined group compared to both APP/PS1 and dbdb models.

**
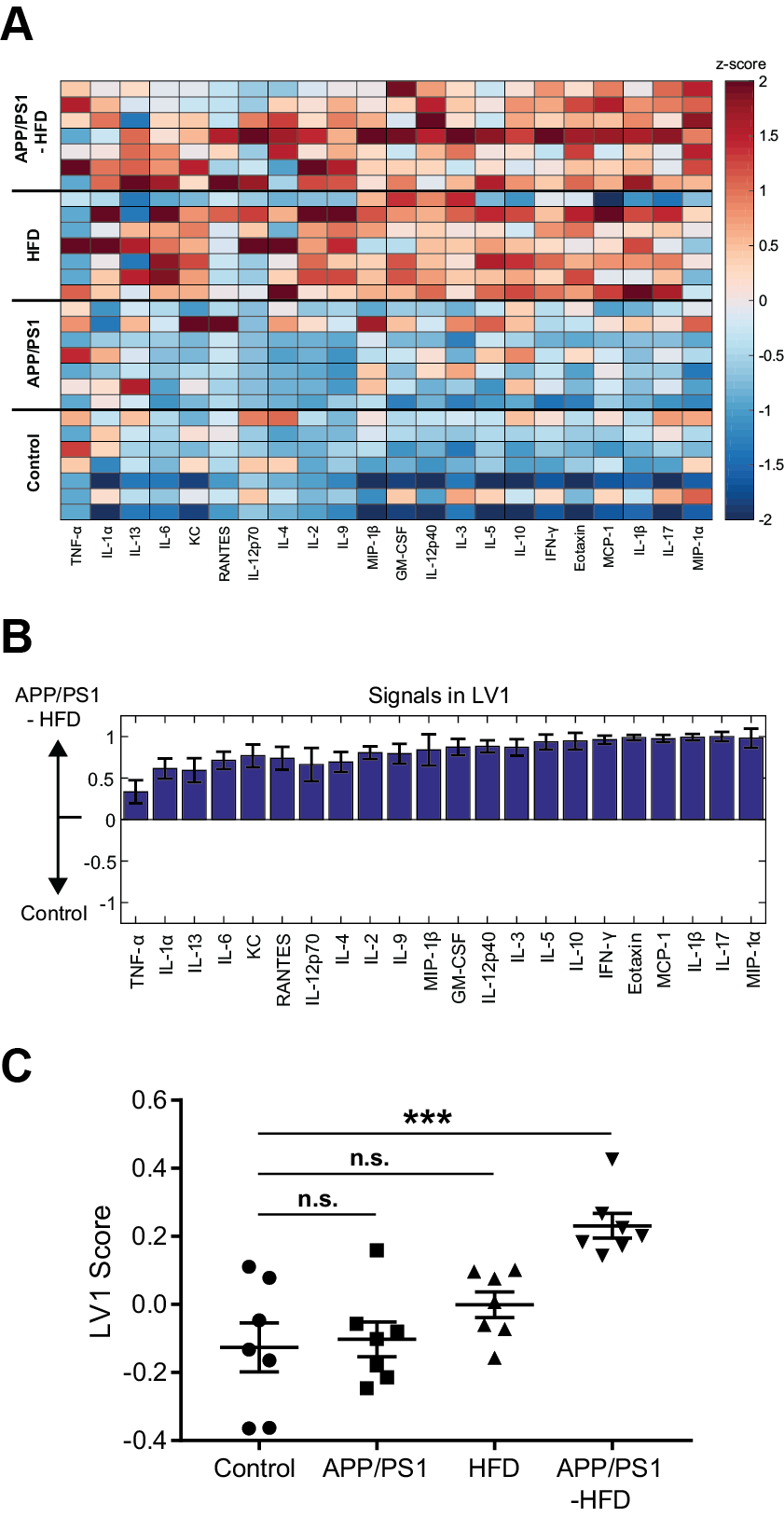
**

**Figure S6. APP/PS1-HFD pathology upregulates a profile of cytokines compared to wild-type controls.**

**A.** Luminex analysis of 22 cytokines (columns, z-scored) expressed in the cortex of control (wild-type), APP/PS1, HFD, and APP/PS1-HFD mice (each row is a cortex sample). **B.** PLSDA identified a profile of cytokines, LV1, that best distinguished APP/PS1-HFD mice (positive) from controls (negative). Errors bars on each cytokine were computed by PLSDA model regeneration using iterative subsampling of 80% of the samples (mean ± SD). **C.** Scoring the data for each sample in A on LV1 revealed that APP/PS1-HFD group is significantly increased on the LV1 cytokine profile compared to control (****p*<0.001; ordinary one-way ANOVA with Dunnett’s test).

**
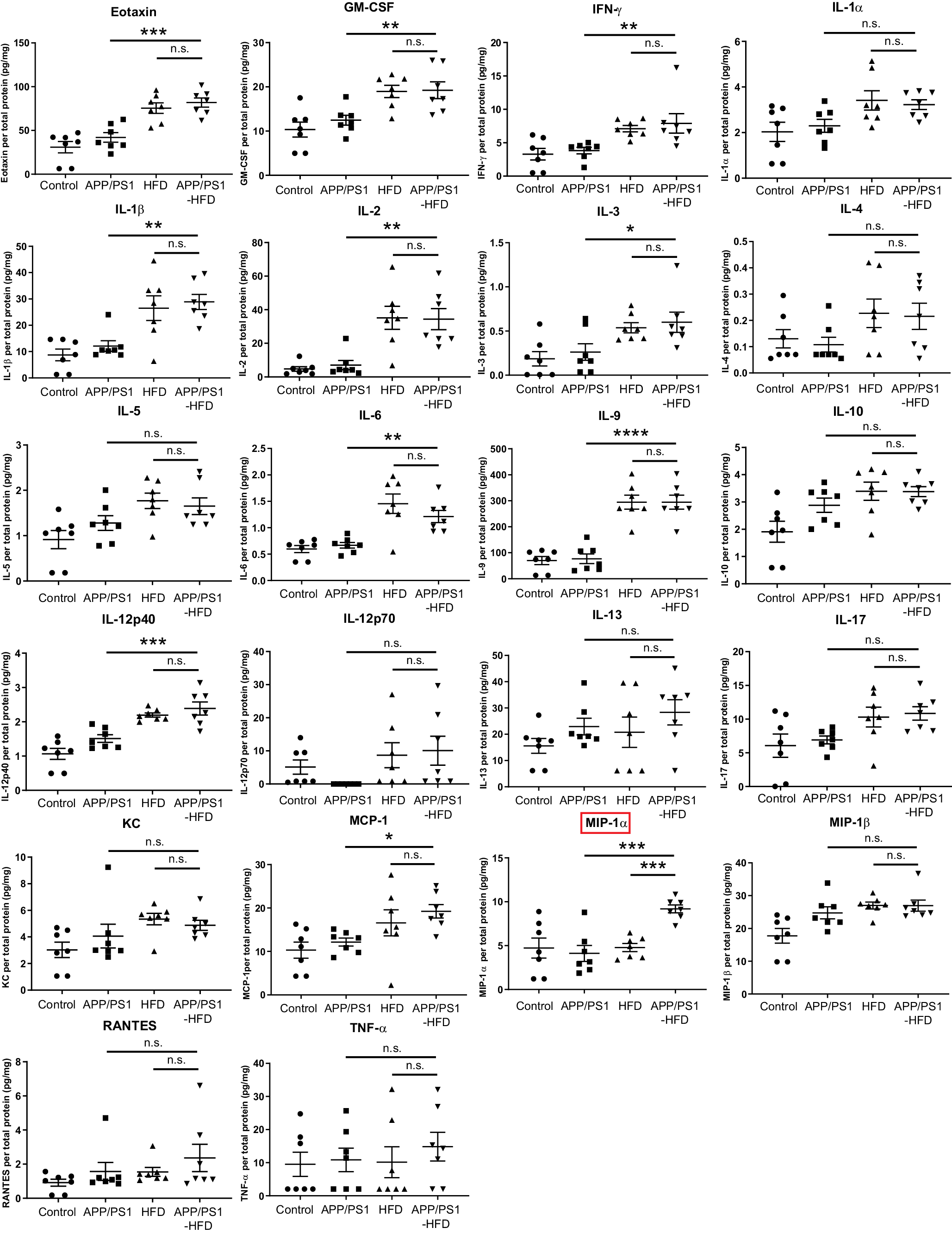
**

**Figure S7. Individual cytokines measured for APP/PS1, HFD, and APP/PS1-HFD groups.**

Bar plots of 22 analytes measured for APP/PS1, HFD, and APP/PS1-HFD groups. APP/PS1-HFD pathology upregulates Eotaxin, GM-CSF, IFN-γ, IL-1β, IL-2, IL-3, IL-6, IL-9, IL-12p40, IL-17, MCP-1, and MIP-1α when compared to APP/PS1, and MIP-1α only when compared to HFD (**p<*0.05, ***p<*0.01, ****p<*0.001, *****p<*0.0001; ordinary one-way ANOVA among APP/PS1, HFD, and APP/PS1-HFD groups with Holm-Sidak’s test). Red box highlights cytokine that is significantly up-regulated in combined group compared to both APP/PS1 and HFD models.
